# Supplementary material for: A set of multi-entry identification keys to African frugivorous flies (Diptera, Tephritidae)
Source: Zookeys. 2014 Jul 24;(428):97–108. doi: 10.3897/zookeys.428.7366 (PMC4143993; doi:10.3897/zookeys.428.7366)
Supplement: Supplementary material 6 — Key to Ceratitis [file zookeys-428-097-s006.zip › SF6_ZooKeys_key to Ceratitis/key/SF6_ZooKeys_key to Ceratitis/Media/Html/Ceratitis brachychaeta.htm]

Ceratitis (Ceratitis) brachychaeta Freidberg


***Ceratitis*** **(*Ceratitis*) *brachychaeta*
Freidberg**

*Ceratitis* (*Ceratitis*) *brachychaeta*
Freidberg, 1991: 169.

Body
length: 3.97 (3.55-4.45) mm; wing length: 3.86 (3.40-4.45) mm.

Male

Head.
Antenna yellow. Third antennal segment twice as long as second segment. Arista
almost bare, at most with very short hairs on base. Frons pale yellow, in
centre darker yellow; with short scattered hairs which are largely the same
colour as frons. Frontal and orbital bristles yellow, ocellar black; lower
orbital modified, flattened with bifurcated end, much shorter than arista;
upper orbital slightly flattened and short but not modified otherwise (missing
in most specimens studied, according original description shortly branched).
Face yellow; lower eye margins sometimes darker yellow. Genal bristle pale,
genal setulae pale, strongly developed. Postocellar and outer vertical bristles
pale.

Thorax.
Postpronotum yellow-white, spot more brownish and weakly developed. Mesonotum ground
colour black-brown, occasionally black, microtrichiae pattern silvery with
ashgrey shine, spots black-brown or black except sutural yellow spots which are
sometimes extended posteriorly as postsutural yellow lines, prescutellar yellow
markings merged. Scapular setae pale. Scutellum yellow-white, basally with two
dark merged spots, apically with three merged spots, incised to halfway.
Anepisternum with lower half darker yellow but not distinct; also hairs on
lower half somewhat darker yellow. 

Legs.
Yellow; setation typical for subgenus, mainly pale especially on femora. Fore
femur posteriorly with bush of longer orange-coloured hairs along entire
length, posterodorsal hairs longer; ventral spines yellow-orange, anteroventral
row of hairs short and yellow-orange. Hind femur with longer hairs dorsally and
ventrally on apical fourth. 

Wing.
Marginal band usually with clear and complete interruption, rarely with very
weak and indistinct bridge; cubital band joined with discal band, sometimes
bridge indistinct or cubital band free; medial band absent; crossvein r-m
beyond middle of discal cell. Vein R1 equal with crossvein r-m. Crossvein dm-cu
posteriorly more outwards than anteriorly.

Abdomen.
Yellow. Setation and banding typical of subgenus.

Female

As
in male except for the following characters: third antennal segment two to
three times longer than second segment. Hairs on arista slightly more
developed. Frons convex to flat, in lateral view very slightly projecting
forwards at antennal implant. Orbitals not modified. All cephalic bristles
dark, except postocellar and outer vertical. Legs without feathering. Wings,
cubital band always free. Oviscape shorter than abdominal terga.

(Description
after De Meyer, 2000)
